# PP2A-Mediated Anticancer Therapy

**DOI:** 10.1155/2013/675429

**Published:** 2013-11-07

**Authors:** Weibo Chen, Zhongxia Wang, Chunping Jiang, Yitao Ding

**Affiliations:** ^1^Department of Hepatobiliary Surgery, Affiliated Drum Tower Hospital of Nanjing University Medical School, Nanjing, Jiangsu 210008, China; ^2^Jiangsu Province's Key Medical Center for Hepatobiliary Surgery, Nanjing, Jiangsu 210008, China

## Abstract

PP2A is a family of mammalian serine/threonine phosphatases that is involved in the control of many cellular functions including protein synthesis, cellular signaling, cell cycle determination, apoptosis, metabolism, and stress responses through the negative regulation of signaling pathways initiated by protein kinases. Rapid progress is being made in the understanding of PP2A complex and its functions. Emerging studies have correlated changes in PP2A with human diseases, especially cancer. PP2A is comprised of 3 subunits: a catalytic subunit, a scaffolding subunit, and a regulatory subunit. The alternations of the subunits have been shown to be in association with many human malignancies. Therapeutic agents targeting PP2A inhibitors or activating PP2A directly have shed light on the therapy of cancers. This review focuses on PP2A structure, cancer-associated mutations, and the targeting of PP2A-related molecules to restore or reactivate PP2A in anticancer therapy, especially in digestive system cancer therapy.

## 1. Introduction

Protein phosphatase 2A(PP2A) is a member of phosphoprotein phosphatase (PPP) family which belongs to the superfamily of protein serine/threonine phosphatases that reverse the actions of protein kinases by cleaving phosphate from serine and threonine residues of proteins. It has been proven that PP2A regulates various cellular processes, including protein synthesis, cellular signaling, cell cycle determination, apoptosis, metabolism, and stress responses [1–3]. PP2A is widely described as a tumor suppressor since the first recognition that its inhibitor okadaic acid is a tumor promoter, and mutations of PP2A subunits can be detected in a variety of human malignancies. The tumor suppressing function of PP2A makes it a possible target in anticancer therapy.

Colorectal cancer is the third most common cancer in males and the second in females, and about 25% of patients with colorectal cancer present with overt metastatic disease. Forty to 50% of newly diagnosed patients can develop metastasis [4, 5]. Liver cancer is the fifth most common cancer in males and the seventh most in females worldwide. It ranks the third in cancer-related deaths [5]. Hepatocellular carcinoma (HCC) which account for 70–85% of primary malignancies in liver is the dominant histological type of primary liver cancer [6]. To date, the treatment of these two cancers is not satisfactory, and the discovery of new therapeutic agents is in demand. Among all the possible targets, PP2A is a promising one.

In this review, we focus on the structure of PP2A and the possible mechanism of its participation in anticancer therapy with special emphasis on targeting PP2A in colorectal cancer and HCC.

## 2. PP2A Structure and Cancer-Associated Mutations

The holoenzyme structure of PP2A comprises a 36 kDa catalytic subunit (PP2AC or C subunit), a 65 kDa scaffolding subunit (PR65 or A subunit), and a regulatory subunit (B subunit). A C subunit and an A subunit make the PP2A core enzyme (PP2AD) which then binds with a B subunit, thus, making the PP2A heterotrimeric holoenzyme (PP2AT).

The catalytic subunit PP2AC is comprised of 309 amino acids and has two different isoforms (*α* and *β*) which are encoded by two separated genes but share 97% sequence similarity. Despite the sequence similarity, PP2AC*α* and PP2AC*β* seem to not be able to compensate for each other because PP2AC*α* knockout mice cannot survive. PP2AC is highly expressed in hearts and brains and is mainly distributed in cytoplasm and nucleus. The regulation of PP2AC is highly organized and precise which is usually made up of phosphorylation at Tyr307 and Thr304 and methylation at Leu309. Phosphorylation at Thr304 is regulated by autophosphorylation-activated protein kinase and can inhibit the recruitment of B55 subunits [7, 8]. Thr307 can be phosphorylated by p60v-src as well as by other receptor and nonreceptor tyrosine kinases which results in a decrease of phosphatase activity and thus can inhibit the interaction with B56 subunits and B55 subunits [9]. The posttranslational modification with methylation at Leu309 is catalyzed by leucine carboxyl methyltransferase 1 (LCMT1) and PP2A methylesterase-1 (PME-1). The methylation can enhance the affinity of PP2A for B55 subunits which can be reversed by phosphorylation at Tyr307 [10] (Table 1).

The A subunit serves as a structural subunit and can bind to a C subunit with its C-terminal repeats 11–15 and to a B subunit with its N-terminal repeats 1–10. The A subunit structure is composed of 15 tandem repeats of a 39 to 41 amino-acid sequence which is called HEAT (huntingtin/elongation/A subunit/TOR) motif. The HEAT repeats of the scaffold A subunit form a horseshoe-shaped fold, holding the catalytic C and regulatory B′ subunits together on the same side [11]. Like the C subunit, the A subunit is also composed of two isoforms (*α* and *β*) which share an 87% sequence similarity, and both are widely expressed in cytoplasm. Despite the consistency in the sequence, the 2 isoforms are functionally distinct and cannot substitute for each other, because overexpressed A*α* fails to revert the transformed phenotype in A*β* suppressed cells [12]. Unlike A*α*, which is ubiquitously expressed in different tissues and cells, the A*β* expression level varies and can sometimes be detected with mutations in tumor tissues with a more common frequency. Mutations of both genes are found to occur at low frequency in human tumors. The gene encoding A*β* was founded to be altered in 15% of primary lung cancers, 15% of colorectal cancers, and 13% of breast cancers, making it unable to bind to B and/or C subunits *in vitro* [13–15]. The alternations include gene deletion, point mutation, missense, and frameshifts. Sablina et al. found that loss of A*β* can permit immortalized human cells to achieve a tumorigenic state and contribute to cancer progression through dysregulation of small guanosine triphosphatase (GTPase) RalA activity which can be dephosphorylated by A*β* at Ser183 and Ser184 and is thus a necessity for the transformed phenotype induced by suppression of A*β* [12, 16]. The A*α* gene alternations can also be found in a variety of neoplasms, like melanomas, breast cancers, and lung cancers, though in a lower frequency when compared with A*β* [14, 17]. To date, 4 kinds of caner-associated mutation of A*α* have been detected: E64D, E64G, R418W, and Δ171-589 [17]. The specific binding of SV40 small t (ST) antigen to A*α* can lead to the elimination of its capacity to from complex with B56*γ* which results in human cell transformation [18]. By introducing A*α* mutants into immortalized but nontumorigenic human cells, Chen et al. found that A*α* mutants can induce functional haploinsufficiency which can somehow lead to the deficiency to dephosphorylate Akt. Then, the active form of Akt results in the human cell transformation [19] (Tables 1 and 2).

The regulatory B subunits are encoded by 4 unrelated gene families: PR55/B (PPP2R2A, PPP2R2B, PPP2R2C, PPP2R2D), PR56/61/B′ (PPP2R5A, PPP2R5B, PPP2R5C, PPP2R5D, PPP2R5E), PR130/72/48/59/G5PR/B′′ (PPP2R3A, PPP2R3B, PPP2R3C), and PR93/110/B′′′ (STRN, STRN3), and each member from the 4 families shows no similarity in sequence. The B family has 4 isoforms: *α*, *β*, *γ*, and *δ*. They all show a time and space expression pattern: the *α* and *δ* isoforms are widely distributed in tissues, while the *β* and *γ* isoforms are enriched in the brain. The expression level of the *β* isoform decreases while *γ* elevates. The mainly subcellular distribution of these 4 isoforms are cytoplasm/nucleus, cytosol, cytoskeletal fraction, and cytosol, respectively [1]. The B′ family has 5 isoforms: *α*, *β*, *γ*, *δ*, and *ε*. Like the B family, they are also expressed and enriched in certain tissues and subcellular cavity. All B′ family members contain a highly conserved central region which is 80% identical and is responsible for the interaction with A/C subunits and a divergent C-terminal and N-terminal which may confer different functions, such as regulation of substrate specificity and subcellular targeting [20]. The B′′ family contains 5 isoforms that might arise from the same gene by alternative splicing. PR 130 is widely expressed in all tissues and enriched in the heart and muscle, while PR72 is exclusively expressed in the heart and muscle. PR48 shares 68% homology with PR59 and is mainly distributed in nucleus. It is an interaction partner of Cdc6 which functions in the initiation of DNA replication. PR59 is believed to be an interaction partner of p107 protein, and when overexpressed, it can bind to and dephosphorylate p107 protein, thus, leading to the expression of DNA damage related genes which induces inhibition of cell cycle progression [21]. The B′′′ family contains newly identified members that share a conserved epitope with the B′ family. PR93, also termed S/G2 nuclear autoantigen (SG2NA), is mainly distributed in the brain and muscle while PR110, also termed striatin mainly in the postsynaptic densities of neuronal dendrites [22]. They both can act as a calmodulin binding protein to interact with PP2AC in a calcium-dependent manner. It is believed that the variety of B subunits accounts for the functional specificity of PP2A. Besides the cancer-associated mutations of the A subunits, the alternations of B subunits can also contribute to cell transformation. Alternation of certain types of B subunits have been detected in neoplasms, like the decreased expression level of B56*γ* in human melanoma cell lines [23]. Ito et al. reported that a truncated B56*γ*1 isoform which can disrupt PP2A phosphatase activity *in vivo* is expressed in a metastatic clone, BL6, of mouse B16 melanoma cells and is sufficient to enhance the metastasis of another clone, F10 [24]. The overexpression of PR65*γ* in human embryonic kidney epithelial cells and human hepatocellular cell lines can revert the cell transformation [18] (Table 1).

## 3. Reactivate PP2A to Augment Anticancer Effect by Targeting Inhibitory Proteins of PP2A

To date, 4 kinds of cellular inhibitory proteins of PP2A has been described, namely, CIP2A, pp32/I_1_^ PP2A ^, SET/I_2_^PP2A^, and SETBP1. Compared with environmental toxins like okadaic acid, they are more selective. Emerging studies suggest that aberrant expression and/or activity of these phosphatase inhibitors may be associated with many human malignancies (Table 3).

### 3.1. CIP2A

The increased expression of cancerous inhibitor of PP2A(CIP2A) has been described in many kinds of malignancy like HCC, breast cancer, colorectal cancer, ovarian cancer, cervical cancer, prostate cancer, lung cancer, chronic myeloid leukemia, and acute myeloid leukemia [25]. In nonsmall cell lung cancer, CIP2A elevation correlated with elevated C-Myc expression levels, and is a significant prognostic predicator for poor survival [26]. Likewise, in acute myeloid leukemia, prostate cancer, and other malignancies, increased CIP2A predicts poor differentiation and worse consequences [27, 28].

Many studies have demonstrated that the disruption and dysfunction of PP2A is a requirement for malignant transformation. Because of PP2A's multiple functions in pathway regulations and variant functions attributed to different PP2A subunits, the mechanism of induced cell transformation is distinct and complicated, such as the dysregulation of Wnt/*β*-catenin signaling pathway and the Bcl-2 family of apoptosis regulators as well as the deficiency in inhibiting the oncogenic transcription factor c-Myc [29]. C-Myc has two phosphorylation residues: Ser62 and Thr58. The phosphorylation of Thr58 permits the dephosphorylation by PP2A on Ser62 which lead to further degradation of C-Myc. In human cell transformation, inhibition of PP2A fails to dephosphorylate c-Myc Ser62, making it overexpressed in malignancies [30]. The possible mechanism underlying the inhibition of PP2A-mediated c-Myc Ser62 dephosphorylation has been interpreted by Junttila et al. The PP2A inhibitor CIP2A can act as the c-Myc stabilizing protein. It can directly bind to c-Myc through recognition of the Ser62 site and then prevent PP2A-dependent dephosphorylation of c-Myc [31].

**Figure 1:**
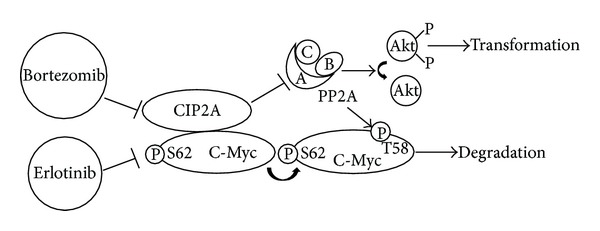
Bortezomib and erlotinib restore PP2A activity by targeting CIP2A. Bortezomib and erlotinib downregulate CIP2A which leads to up-regulation of PP2A activity and therefore inhibiting cell transformation by inactivating Akt.

In consideration of its inhibition of PP2A in the stabilization of c-Myc and other signals like the Akt signal, CIP2A has be found to be an anticancer target. CIP2A is reported to be a target of bortezomib in many kinds of malignancies, such as HCC, leukemia, human triple negative breast cancer, and head and neck squamous cell carcinoma [32, 33]. Chen et al. found that bortezomib can downregulate CIP2A in a dose- and time-dependent manner, and can upregulate PP2A activity in HCC. The inhibition of CIP2A by bortezomib leads to PP2A-dependent Akt inactivation and tumor cell apoptosis [34]. The apoptotic effect of bortezomib is also described in leukemia cells by downregulation of CIP2A and upregulation of PP2A activity [35]. Bortezomib can sensitize solid tumor cells to radiation through the inhibition of CIP2A [36]. Besides apoptosis-inducing role of bortezomib by antagonizing CIP2A, the induced autophagy by bortezomib also depends on the down-regulation of CIP2A and p-Akt in HCC [37]. And in sensitive hepatocellular cells, the apoptosis- inducing effect of erlotinib is mediated by down-regulation of CIP2A besides its status as a selective epidermal growth factor receptor (EGFR) tyrosine kinase inhibitor (TKI). The newly discovered effect of erlotinib by CIP2A-dependent p-Akt down-regulation makes CIP2A a possible target in the treatment of HCC [38] (Figure 1).

### 3.2. PHAPI/pp32/**I_1_^PP2A^**

The protein putative human HLA-DR-associated protein I(PHAPI), which has been variously identified as pp32 or I_1_^PP2A^, is a putative HLA class II-associated cytosolic protein and also a potent tumor suppressor. It has been shown to be a PP2A inhibitor though the detailed mechanism has not been fully understood, probably by binding directly to the C subunit [39]. Yu et al. found that the antiproliferative lectin, jacalin, can dissociate PP2A from PHAPI through inducing tyrosine phosphorylation of PHAPI in HT29 colon cancer cells [40]. This may seem contradictory because PHAPI itself is a tumor suppressor and PP2A is also a tumor suppressor. However, no proper explanation has been discovered. The inhibitory function of PHAPI is probably dominant in the aspect of inducing apoptosis.

### 3.3. SET/**I_2_^PP2A^**

The oncogene SET and its truncated cytoplasmic form I_2_^PP2A^ are also inhibitory proteins of PP2A. It is discovered that SET is fused with the nucleoporin NU214 (CAN), and it is associated with myeloid leukemogenesis and highly expressed in Wilms' tumors and BCR-ABL1-positive leukemia. Its overexpression predicts poor prognosis [41]. Elevated expression of SET has been linked to cell growth and transformation. SET can inhibit PP2A by forming an inhibitory protein complex with PP2A [39]. Besides, it can also form an inhibitory complex with nm23-H1 which can inhibit tumor metastasis [42].

Eichhorn et al. discovered that a novel apolipoprotein E-based peptide, COG112, can inhibit the interaction of SET with PP2AC, leading to increased PP2A activity. With increased PP2A activity, the p-Akt and c-Myc activity decreases. COG112 can also release SET from nm23-H1, thus, restoring the metastasis suppressor function of nm23-H1 [43]. Also, apolipoprotein E-mimetis peptides can bind to SET, therefore, restoring PP2A activity [44].

### 3.4. SETBP1

The SET binding protein (SETBP1) is a SET regulator, and it is fused in frame with a nucleoporin, NUP98. SETBP1 is overexpressed in 27.6% of acute myeloid leukemia at diagnosis and is associated with poor prognosis, particularly in elderly patients, as patients with SETBP1 overexpression had a significantly shorter overall survival and event-free survival in patients over 60 years. SETBP1 overexpression protects SET from protease cleavage which increases the amount of full-length SET protein and leads to the formation of a SETBP1-SET-PP2A complex. The SETBP1-SET-PP2A complex can inhibit PP2A and therefore promote the proliferation of leukemic cells [45, 46]. Piazza et al. found mutated SETBP1 (encoding a p.Gly870Ser alternation) to be a new oncogene present in atypical chronic myeloid leukemia as cells expressing this mutant exhibit higher amounts of SETBP1 and SET protein and lower PP2A activity [47].

## 4. PP2A-Activating Drugs

PP2A can be activated by various agents though direct or indirect interaction, like ceramide and FTY720. The general consequence of reactivation of PP2A in malignancies is apoptosis of cancer cells, and others may include cell proliferation inhibition and cell cycle arrest.

### 4.1. Ceramide

Ceramide has been shown to be a potent tumor suppressor which can trigger apoptosis and autophagy and limit cancer cell proliferation. The downstream of ceramide include many players, like PP2A, p38, JNK, Akt, and survivin. In ceramide-induced mitochondrial outer membrane permeabilization (MOMP), which is a key event in apoptotic signaling, the activation of PP2A is an important step to activate GSK-3*β*. Ceramide-activated PP2A can dephosphorylate GSK-3*β* at Ser9 through PI3K-Akt pathway [48]. In ceramide-induced cell cycle arrest, the accumulation of p27 is due to the activation of PP2A which leads to the inhibition of Akt [49].

### 4.2. FTY720

The immunosuppressant FTY720 is a sphingosine analogue that is approved for the treatment of relapsing multiple sclerosis. It can induce apoptosis in peripheral blood lymphocytes. Its anticancer function has been discovered in many kinds of malignancies, like breast cancer, leukemia, HCC, and prostate cancer, and so forth [50, 51]. However, the mechanism underlying the anticancer therapy varies. In HCC cells, the FTY720-induced apoptosis is mediated through the PKC*δ* signal. In leukemia cells, FTY720-direct mitochondrion-related apoptosis is mediated by FTY720-induced PP2A activation which is the outcome of FTY720 disrupting SET-PP2A interaction [51, 52].

Other PP2A-activating drugs, like forskolin, chloroethylnitrosourea, and vitamin E analogues, and so forth, are listed in Table 4. A PP2A-activating protein E1A is also described. It is reported to increase PP2A activity by upregulating PP2AC subunits, which results in the repression of Akt activation and the subsequent apoptosis [53].

## 5. Targeting PP2A in Digestive System Cancers

The PP2A alternations and PP2A inactivation have been described in many kinds of digestive system cancers, like colorectal cancer and HCC. Suppression of PP2A activity may serve a carcinogenesis role in alimentary system malignancies. PP2A subunits have been found to be mutated or deleted to some degree in various digestive system cancers. The subunit A*β* alternation has been detected in 15% of colorectal cancer, and it is related to its capacity of binding to a B and/or a C subunit, which leads to a decreased PP2A activity [13]. Besides the relatively low frequency of PP2A mutations and deletions, Tan et al. found that the epigenetic mechanism may play a dominant role in PP2A inactivation in colorectal cancer. They found that epigenetic silencing of PP2A regulatory B55*β* subunit can be detected in more than 90% of colorectal cancers [71]. The PP2A inhibitory protein CIP2A is increased in colorectal cancer and HCC, accompanied by impaired PP2A activity. So, in digestive system cancers, PP2A has its unique role in malignancy suppression and can be a target in anticancer therapy.

In colon cancers, researches reveal that resistance to antiangiogenesis therapy exists in CSLCs. It is the consequence of PP2A activity suppression in CSLCs in colon cancer cells. By suppressing PP2A, the p38 mitogen-activated protein kinase (MAPK) pathway is activated, which leads to the activation of (heat shock protein 27) Hsp27 and the subsequent antiapoptotic effect of Hsp27 [72]. PP2A inhibition in CSLCs of glioblastoma effectively controls the cell differentiation and/or death through modulating the Akt/mammalian TOR (mTOR)/GSK-3*β* pathway [73]. In colorectal cancer, PP2A inhibition is essential for the maintenance of CSLCs through the Akt Ser473/mTOR pathway [74]. So, the inhibition of PP2A confers CSLCs the characteristics of stem cells and is of significant importance to the initiation of cancers. The therapeutic reactivation of PP2A in CSLCs can be a possible anticancer treatment against drug resistance and recurrence. Wang et al. did find that by activating PP2A, silibinin can suppress the self-renewal of CLSCs and inhibit sphere formation and tumor initiation in colorectal cancer [74].

As has been mentioned, ceramide is a potent tumor suppressor which can lead to tumor cell apoptosis and autophagy. By activating PP2A, ceramide can induce apoptosis and cell cycle arrest either by GSK-3*β* activation or p27 accumulation. Ceramide is a sphingolipid consisting of sphingosine, and sphingadienes (SDs) is another derivative from soy and other natural sphingolipids. SDs are reported to inhibit cell growth and tumorigenesis by inhibiting Wnt signaling through PP2A/Akt/GSK-3*β* pathway in colon cancer [75]. The chemopreventive effect of SDs relies on PP2A activation, which may serve as an upstream target of SDs in downregulating Wnt signaling. The activating mutations in Wnt signaling have been linked to the initiation of colorectal cancer [76]. However, the down-regulation of Wnt signaling by PP2A is not universal. Aspirin and mesalazine are found to be able to decrease Wnt/*β*-catenin in colorectal cell lines which make them possible chemopreventive agents. Aspirin and mesalazine treatment are both associated with phosphorylation of PP2A which is an inactive form of PP2A [77, 78]. Some other anti-colon-cancer agents like dihydroxyphenylethanol (DPE) are also reported to induce apoptosis or cell cycle arrest by activating PP2A [79].

In HCC, as mentioned above, CIP2A overexpression can be detected, and bortezomib can downregulate CIP2A and upregulate PP2A activity in HCC. The inhibition of CIP2A by bortezomib leads to tumor cell apoptosis and autophagy. HCC cells with high levels of CIP2A are more resistant to bortezomib treatment than those with low level of CIP2A. Erlotinib can also downregulate CIP2A which leads to apoptosis in HCC. A few erlotinib derivatives have been found as CIP2A-ablating agents in HCC cell line SK-Hep-1. The compounds N4-(3-Ethynylphenyl)-6,7-dimethoxy-N2-(4-phenoxyphenyl) quinazoline-2,4-diamine and N2-Benzyl-N4-(3-ethynylphenyl)-6,7-dimethoxyquinazoline-2,4-diamine can induce HCC cell apoptosis by inhibiting CIP2A [80]. *Zanthoxylum avicennae* extracts (YBBEs) and diosmin can inhibit HCC cell line HA22T cell proliferation by activating PP2A [81, 82].

## 6. Conclusion

It has been almost 30 years since the first recognition that okadaic acid is a tumor promoter and targets PP2A [83], and emerging studies have made it solid that PP2A is a tumor suppressor and that its regulation can be a target for anticancer therapy. This review is mainly focused on the restoration and activation of PP2A in human malignancies by targeting PP2A inhibitory proteins or directly activating or upregulating PP2A. However, we should never neglect the controversy that exists that whether PP2A is a real tumor suppressor, because there are also ever-growing evidences against this fundamental hypothesis. For example, Zimmerman et al. found that the inactivation of PP2A, in particular, of the B56*γ* and B56*δ* subunits, is a crucial step in triggering apoptin-induced tumor-selective cell death [84]. Antitumor drugs like cantharidin control cell cycle and induce apoptosis by inhibiting PP2A [85], and cell viability inhibition and proapoptotic effect of cantharidin in PANC-1 pancreatic cancer is mediated by the PP2A/I*κ*B kinase (IKK*α*)/I*κ*B*α*/p65 NF-*κ*B pathway [86]. Accordingly, PP2A-mediated anticancer therapy may include two opposed aspects, activation and inhibition, mainly depending on the cell types or the transforming agents. And despite the progress made in the field of targeting PP2A in anticancer therapy, there is still a long way ahead to clinical application. Much effort is needed in the molecular mechanisms and medical translation of possible therapeutic agents targeting PP2A.

## Figures and Tables

**Table 1:** Nomenclature of subunits of PP2A and the subcellular distribution.

Subunit	Gene name	Gene locus	Isoforms	Aliases	Subcellular distribution	References
A	PPP2R1A	19q13.14	A*α*	PR65*α*	Cytoplasm	[1, 25, 54, 55]
	PPP2R1B	11q23.1	A*β*	PR65*β*	Cytoplasm	[1, 13, 25, 54]

B	PPP2R2A	8p21.1	B*α*	PR55*α*, B55*α*	Cytoplasm, microtubules, neurofilaments, vimentin, membrane, nucleus, Golgi/reticulum	[1, 25, 54]
	PPP2R2B	5q32	B*β*	PR55*β*, B55*β*	Cytosol	[1, 25, 54, 56]
	PPP2R2C	4p16.1	B*γ*	PR55*γ*, B55*γ*	Cytoskeletal fraction	[1, 25, 54, 57]
	PPP2R2D	10q26.3	B*δ*	PR55*δ*, B55*δ*	Cytosol	[1, 25, 54]

B′	PPP2R5A	1q32.3	B′*α*	PR61*α*, B56*α*	Cytoplasm	[1, 25, 54]
	PPP2R5B	11q13.1	B′*β*	PR61*β*1, B56*β*, PR61*β*2	Cytoplasm	[1, 25, 54, 58]
	PPP2R5C	14q32.31	B′*γ*1 B′*γ*2 B′*γ*3	PR61*γ*1, B56*γ*1, B′*α*3 PR61*γ*2, B56*γ*2, B′*α*2 B56*γ*3, B′*α*1	Cytoplasm, nucleus, focal adhesion	[1, 25, 54]
	PPP2R5D	6p21.1	B′*δ*	PR61*δ*, B56*δ*	Cytosol, mitochondria, nucleus, microsomes	[1, 25, 54]
	PPP2R5E	14q23.2	B′*ε*	PR61*ε*, B56*ε*	Cytoplasm	[1, 25, 54]

B′′	PPP2R3A	3q22.1	B′′*α*1 B′′*α*2	PR130 PR72	Centrosome and Golgi Cytosol	[1, 25, 54, 59]
	PPP2R3B	Xp22.33	B′′*β*1 B′′*β*2	PR48 PR59	Nucleus	[1, 25, 54]
	PPP2R3C		B′′*γ*	G5PR	Nucleus	[1, 25, 54]

B′′′	STRN	2p22.2		PR110, PR93	Membrane and cytoplasm	[1, 25, 54]
	STRN3	14q12		PR112, PR102,PR94	nucleus	[1, 25, 54]

C	PPP2CA	5q31.1	C*α*	PP2A*α*	Cytoplasm and nucleus	[1, 25, 54]
	PPP2CB	8p12	C*β*	PP2A*β*	Cytoplasm and nucleus	[1, 25, 54, 60]

**Table 2:** Cancer-associated mutations of PP2A A subunits.

Subunit	Mutation name	Alternations	Consequence	Cancer type	References
PR65*α*	E64D E64G R418W	Point mutation	Deficiency in binding to B′*α*1 Deficiency in binding to B/C subunits	Breast Lung Skin	[15, 17, 61]
	Δ171-589	Deletion		Breast	

PR65*β*	G8R P65S G90D L101P L101P/V448A K343E V448A D504G V545A	Missense	Dysregulation of RalA GTPase, leading to impaired binding capacity to B/C subunits	Lung Breast Colon	[12, 15, 43]
	ΔE344-E388	In-frame deletion			[12, 15, 43]

**Table 3:** Inhibitory protein of PP2A and possible related anticancer drugs.

Inhibitory protein	Interaction with PP2A	Drugs against inhibitory	References
CIP2A	Prevent PP2A-dependent dephosphorylating of c-Myc	Bortezomib; Erlotinib	[32–35]
PHAPI/pp32/I_2_^PP1A^	Direct binding	Jacalin	[39, 40]
SET/I_2_^PP2A^	Direct binding	COG112; Apolipoprotein E-mimetis peptides	[39, 43, 44]
SETBP1	Form a SETBP1-SET-PP2A complex		[45]

**Table 4:** PP2A-activating drugs/protein.

Activating drugs/Proteins	Mechanism	Malignancies	Consequences	References
Ceramide	Activation of PP2A, leading to activation of GSK-3*β* or accumulation of p27	Prostate cancer	Apoptosis; Cell cycle arrest	[48, 49]
FTY720	Disrupt SET–PP2A interaction	Leukemia	Apoptosis	[51, 52]
Forskolin	Induces PP2A activity by increasing intracellular cAMP levels	Leukemia	Block proliferation; induce apoptosis	[62, 63]
Chloroethylnitrosourea	Augment methylation of PP2A, leading to Akt dephosphorylation	Melanoma	Reduce cell proliferation and survival	[64, 65]
Vitamin E analogues (i.e., *α*-tocopheryl succinate)	Reduce PKC*α* isotype (colon cancer) or inactivation of JNKs (prostate cancer) activity by increasing PP2A activity	Colon, prostate cancer	Apoptosis	[66, 67]
Carnosic acid	Downregulate AKT/IKK/NF*κ*B by activation of PP2A	Prostate cancer	Apoptosis	[68]
Methylprednisolone	Upregulate PP2A B subunits	Myeloid leukemia	Cell differentiation	[69]
Dithiolethione	Increase PP2A concentration	Lung, breast cancer	Inhibit proliferation	[70]
E1A	Upregulate PP2A C subunits	Breast cancer	Apoptosis	[53]
